# Oral Mucosal Human Papillomavirus and Epstein-Bar Virus Rates in Patients with Dry Mouth and/or Sjögren’s Syndrome in a Hungarian Cohort

**DOI:** 10.3290/j.ohpd.b5718350

**Published:** 2024-08-29

**Authors:** Csilla Erdei, Ágnes Heizer, Károly Mensch, Krisztina Szarka, Emese Virág Kiss, Krisztina Márton

**Affiliations:** a Assistant Professor, Department of Preclinical Dentistry, Faculty of Dentistry, Semmelweis University, Budapest, Hungary. Idea, hypothesis, experimental design, performed the experiment, performed a certain test, wrote the manuscript, consulted and performed statistical evaluation.; b Dentist, Department of Preclinical Dentistry, Faculty of Dentistry, Semmelweis University, Budapest, Hungary. Performed the experiment, proofread the manuscript.; c Lecturer, Department of Oral Diagnostics, Faculty of Dentistry, Semmelweis University, Budapest, Hungary. Idea, hypothesis, experimental design, proofread the manuscript.; d Associate Professor, Institute of Metagenomics, University of Debrecen; Debrecen, Hungary. Experimental design, performed the experiment, wrote the manuscript.; e Professor, Director, National Institute of Locomotor System Diseases and Disabilities, Department of Locomotor System and Rheumatology Prevention, Semmelweis University; Budapest, Hungary. Idea, hypothesis and experimental design.; f Professor, Head, Department of Preclinical Dentistry, Faculty of Dentistry, Semmelweis University, Budapest, Hungary. Idea, hypothesis, experimental design, performed the experiment, wrote the manuscript, consulted and performed statistical evaluation. Proofread the manuscript.

**Keywords:** dry mouth, Epstein–Barr virus, human papillomavirus, oral mucosa, Sjögren’s syndrome

## Abstract

**Purpose:**

To find an association between oral mucosal human papilloma- and/or Epstein–Barr (HPV, EBV) virus infection in patients with dry mouth and/or Sjögren’s syndrome (SS) compared to healthy controls and to find connections with salivary gland histopathological alterations.

**Materials and Methods:**

Ninety-two participants were divided into four groups: 1. healthy controls (n = 32); 2. xerostomia (n = 28); 3. hyposalivation (n = 22); and 4. SS groups (n = 10). To detect virus infection brush biopsy was outlined in all groups. Detections of virus-specific sequences were achieved with polymerase chain reaction (PCR). Lip biopsy and histopathological assessment was performed in groups 2, 3 and 4.

**Results:**

HPV positivity of oral mucosal cells was shown in group 1: 1 (3.12%); group 2: 3 (10.7%); group 3: 2 (8.26%); and in group 4: 0 of the samples. EBV was present in group 1: 14 (43.7%); group 2: 17 (60.7%); group 3: 6 (27.3%); and in group 4: 5 (50%) of the cases. There was no statistically significant difference between the attributes. Intact salivary gland in 28.2%, chronic sialadenitis in 28.2%, stromal fibrosis in 6.5%, lipomatous atrophy in 8.6%, fibrous atrophy in 6.5% and positive focus score (SS) in 26.1% were found in the subjects. Neither HPV nor EBV infection caused statistically significantly more histological abnormalities.

**Conclusion:**

Orofacial mucosal HPV and/or EBV DNA rates did not differ statistically significantly in patients with xerostomia or hyposalivation or SS compared to healthy controls, therefore, it cannot prove the provocative role of these viruses in dry mouth and/or SS. Neither dry mouth nor SS were accompanied by statistically significantly more salivary gland alterations in HPV- and/or EBV-positive subjects; these alterations are frequent in the virus-negative cases too.

The sensation of dry mouth – xerostomia – is a subjective feeling.^21,31^ Nevertheless, it is considered objectively – as hyposalivation – when the flow rate of unstimulated whole saliva is decreased to 0.1 ml/min or less. However, patients may report dry mouth even with normal secretion rates. Hyposalivation is accompanied by deficiency in the protective function of saliva: reduced antimicrobial effect and a decrease in salivary washing function, making it easier for pathogens to infect and colonise oral mucosa. It can also be expected that lesions in the oral mucosa occur more frequently in these patients.

Dry mouth is mostly a symptom of systemic conditions: a side effect of different drugs, ageing, diabetes, radiotherapy of the head and neck region, and is frequent when there is focal inflammation in the salivary glands, namely in Sjögren’s syndrome. Primary Sjögren’s syndrome is a systemic autoimmune disease of unknown aetiology, characterised by the inflammatory dysfunction of the exocrine glands, resulting in decreased function of the salivary and lacrimal glands. Arthralgia, vasculitis, and hypothyroidism are often among the extra glandular symptoms. Sjögren patients often show up with xerostomia as the first sign of the disease in the dental office.^7^ Nevertheless, dry mouth and dryness of the eyes without the serological and/or glandular inflammatory signs of the autoimmune disease is considered a ‘Sicca syndrome’ diagnosis without definitive Sjögren’s syndrome. Sicca patients might have unpleasant orofacial dryness symptoms, like burning mouth, glossodynia and dysphagia. These signs might reveal on one hand the harmfulness, and on the other hand the susceptibility of the oral mucosa to viral, bacterial, and fungal infections that might have the potential to provoke other systemic or local diseases.^22,31^

Several studies support that viral pathogens like EBV, human T-cell lymphotropic virus, parvovirus B19, rubella virus, human immunodeficiency virus (HIV),^29^ herpes simplex virus, hepatitis B virus, hepatitis C virus and cytomegalovirus play key roles in the pathogenesis of autoimmune diseases, such as rheumatoid arthritis, systemic lupus erythematosus, multiple sclerosis and Sjögren’s syndrome, although the precise viral initiating event is still unknown. Genetic inheritance and environmental factors were also suggested pathogenesis of primary Sjögren’s syndrome; however, the aetiology remains unclear. Nevertheless, viral and bacterial (*Mycobacterium* non-tuberculosis-related type, *Helicobacter pylori*) infections are considered as significant external factors in the aetiology, because of their chronic inflammatory effect.^13,24^ The triggering mechanism might be the inflammation via the infection, and the consequent functional impairment of the affected organ and, furthermore, the over-stimulated immune system. Among the viruses, the hepatitis C virus has been considered as one of the exogenous factors in the aetiopathogenesis of Sjögren’s syndrome.^14^ Fox and Fox assert that viral particles might function as autoantigens, which appeal to auto-antibodies generating the proliferation of B cells. Infected mucosal cells may also activate cytotoxic T cells in response, with the help of the major histocompatibility class I-antigen presentation pathway. These attacks might provoke autoimmune diseases. They emphasise that the human T-lymphocytic virus type-1 and type-5, the Epstein–Barr virus, and the Coxsackie virus also play an important role in activating auto-inflammation. Several other infecting agents, including human herpes virus,6 HIV, and hepatitis B have also been reported to trigger primary Sjögren’s syndrome.^9^

Among viruses, the human papillomavirus (HPV) and Epstein–Barr virus (EBV) might also infect epithelial cells.^3,6,12^ Therefore, it might be expected that a decrease in saliva production may increase the susceptibility of the mucosa to HPV and/or EBV infections.

In our study, the HPV virus group encompasses a small-sized double-stranded DNA virus with nearly 130–220 known different subtypes.^3,11,32^ Diseases caused by HPVs in the oral mucosa can either be benign lesions or invasive neoplasms. Malignant tumours are associated with genotypes with a high oncogenic risk (HPV-16 and 18),^32^ while those with a low oncogenic risk (HPV-6 and 11) are linked mainly, with benign lesions.^3,21^

Human papillomaviruses (HPVs) are conveyed via sexual or direct contact with injured skin surface, invading epithelial cells and/or the mucosal tissues.^33^ It is one of the most common sexually transmitted infection and, it is also the cause of most anogenital cancers. Ninety per cent (90%) spontaneously resolve over 3 years, however, and, about 10% remain as persistent infection in the cervical mucosa. Kim et al hypothesised that immunosuppressed individuals are more likely to have HPV persistence, which is necessary for malignant transformation. Accordingly, women with autoimmune diseases are likely vulnerable to HPV infection and the progression of cervical disease.^15^ Majewski and Jablonska found that HPV could serve as super antigens to activate polyclonal T cells, which could trigger autoimmune phenomenon of psoriasis.^18^

Chen et al followed 47,302 patients with first HPV diagnoses and 189,200 non-HPV controls for 12 years. During the follow-up period, the adjusted hazard ratio of primary Sjögren’s syndrome in patients with HPV infections showed to be significantly higher than in the controls, independently from age and gender.^4^

HPV has a principal role in oral squamous cell cancer causing high mortality in Central Eastern Europe and, especially, in Hungary.^5,28^ Head and neck cancers, particularly those of the oropharyngeal region are HPV-associated cancers, with over 90% HPV-16 positivity, so it may be important to know whether dry mouth and/or Sjögren’s syndrome increases the incidence of HPV infection.

EBV is a member of the gamma herpesvirus family and was identified as the first human ‘tumour virus’. In the presence of severe immunosuppression, EBV infection can lead to the development of various lymphomas and neoplasms of epithelial origin. In addition to its association with human cancer, EBV may also be concomitant with the development of other, non-malignant conditions, such as autoimmune diseases.^6,20^

The role of EBV as a cofactor leading to immune dysfunction has been proposed in multiple sclerosis (MS), systemic lupus erythematosus (SLE), rheumatoid arthritis, autoimmune thyroiditis, liver diseases, inflammatory bowel diseases, myasthenia gravis and insulin-dependent diabetes mellitus. There is also a growing body of literature on the association of xerostomia, including Sjögren’s syndrome.^10^

Several factors seem to support the role of EBV in the pathogenesis of reduced saliva production and ultimately in Sjögren’s syndrome: (i) EBV may be present in the whole salivary gland tissue, as their epithelial cells are also the sites of viral latency, and they emphasise that an increased immune response to EBV may cause damage to the structure of salivary glands; (ii) EBV DNA is present in significantly higher copy numbers in salivary gland biopsies from patients with Sjögren’s syndrome compared to healthy salivary glands, suggesting that the immune system is unable to control EBV replication; (iii) there is increased expression of human leukocyte antigen (HLA)-DR in salivary epithelial cells from patients with Sjögren’s syndrome and presentation of EBV-like antigens to T cells. In summary, therefore, salivary gland damage is presumably the result of chronic EBV infection, inadequate EBV-specific immune response and interference with host immunological processes (eg, antigenic mimicry, EBV microRNA, viral IL-10, perturbation of epigenetic regulatory processes).^17^

The results of two research groups based on seroprevalence studies also support the link between EBV and Sjögren’s syndrome.^2,34^ Both groups compared EBV serological patterns in Sjögren’s syndrome patients with those of healthy controls. Barcelos and colleagues (2021) also compared serological markers with serological results in patients with rheumatoid arthritis (RA). Their results were similar; IgG positivity for EBV early antigen was significantly higher than in healthy controls, while the overall seropositivity in the study populations was around 80%. Xuan and his team (2020) also performed a meta-analysis of additional literature data; the analysis showed that, in addition to EBV anti-Early Antigen (anti-EA) IgG positivity, anti-Viral Capsid Antigen (anti-VCA) IgM positivity rates were also significantly higher in patients with Sjögren’s syndrome, which may be indicative for the reactivation of EBV infection.^34^ Studies by Barcelos and colleagues (2021) also shed light on T-cell processes in EBV-associated autoimmune diseases; they found that EBV-specific CD8+ T-cell count increased during B-cell transformation and during the productive replication phase of EBV in patients with EBV-associated RA and SLE.^2^

It is not known whether the oral mucosal HPV and/or EBV infections are more frequent in Hungarian patients with orofacial sicca symptoms and/or Sjögren’s syndrome and if these viruses might have a possible role in provoking the autoimmune systemic inflammation resulting in Sjögren’s syndrome. These viruses’ possible connection to histopathological alterations in the salivary glands is not either known. Investigating the possible viral infection of the oral epithelium in patients with dry mouth may provide information on whether dry mouth and/or Sjögren’s syndrome, or salivary gland histopathological changes have an association with HPV and/or EBV infections.

The aim of this present study was to investigate the prevalence of oral mucosal human papillomavirus and additionally Epstein–Barr virus infection by collecting exfoliated cells from the entire oral mucosal surface of patients with dry mouth (xerostomia and/or hyposalivation), and/or Sjögren’s syndrome compared with the positivity rate of healthy controls. A further aim was to determine the viral infection’s possible associations with these conditions.

Another aim was to find a connection between the possible viral infections of the oral mucosa and the histopathological findings of the minor salivary gland biopsies in the same patient groups.

## MATERIALS AND METHODS

Sixty patients (54 female, 6 male; mean age 56.7 ±15.6 years) with xerostomia and/or xerophthalmia were referred to the ‘Xerostomia Clinic Working Group’ (Teaching Centre, Faculty of Dentistry, Semmelweis University, Budapest, Hungary) between January 2021 and December 2021 in order to confirm primary Sjögren’s syndrome; 32 healthy controls (29 female, 3 male; mean age 49.7 ± 8.7 years) were also evaluated.

Patients presented from different institutes, clinics and dental offices of Central Hungary and from the capital, Budapest: the National Institute of Locomotor System and Disability, the Buda Hospital of the Hospitaller Order of Saint John of God, the Department of Ophthalmology, the Department of Internal Medicine and Haematology and the Department of Dermatology of Semmelweis University, the Medicover Eiffel Clinic, from regionally competent general dental practitioners and from General Practitioners. The main complaints of the subjects were dry eyes and/or dry mouth, rheumatic joint inflammations, and arthralgia. Most of them had at least one treated systemic disease (eg, hypertonia, diabetes mellitus, heart disease, etc.) and took different medications for the systemic disease treatment. Diagnosis of primary Sjögren’s syndrome was confirmed by the ACR-EULAR Diagnostic System (2016).^27^ Subjects who had previously undergone radiotherapy, chemotherapy, immunosuppressive therapy or HPV vaccination were excluded. Age- and sex-matched healthy control persons were completely healthy, and none of them took any medications at the time of their referral. All of them came for routine dental treatment to the Teaching Centre of the Dental Faculty, Semmelweis University.

The study was approved by the Regional Institutional Scientific and Research Ethics Committee of Semmelweis University (SE RKEB 132/2020). All participants of the recent study were provided with an informed written consent form.

### Assessment of the Subjective Orofacial Sicca Symptoms

After a detailed general and dental history and stomato-oncological screening participants were provided with a questionnaire to clarify and specifically define the subjective orofacial and ophthalmological symptoms. As part of the ACR-EULAR diagnostic criteria, the following questions were asked:

1. Have you had daily, persistent troublesome dry eyes for more than 3 months?
2. Do you have a recurrent sensation of sand or gravel in the eyes?
3. Do you use tear substitutes more than three times a day?
4. Have you had a daily feeling of dry mouth for more than 3 months?
5. Do you frequently drink liquids to aid in swallowing dry foods?^27^

If the patient answered ‘yes’ to any of the questions, the examination continued with the assessment of the objective symptoms.

### Measurement of Unstimulated Whole Saliva (UWS) Flow Rate

UWS flow rate was measured by sialometry and was expressed in ml/min.^19^

### Minor Salivary Gland Biopsy

Minor salivary gland biopsy sampling was performed in 46 cases.^1^ Histopathological examination following sampling was completed at the Department of Pathology and Experimental Cancer Research, Faculty of Medicine, Semmelweis University. After fixation and haematoxylin-eosin staining samples 2–4 μm were evaluated at several magnifications (40× to 250×).

#### Cytology sampling of exfoliated cells of oral mucosa

**Fig 1:**
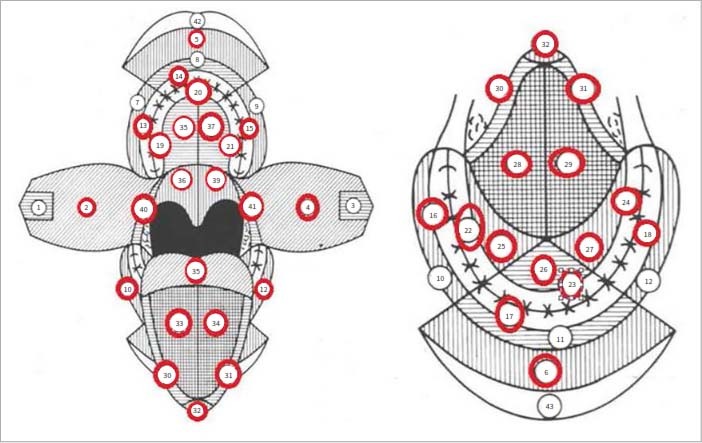
Order of exfoliative sampling from representative areas of the oral cavity according to Mensch et al.^2^

Exfoliative human papillomavirus and Epstein–Barr virus screening were accomplished using brush biopsy sampling based on the method of Mensch et al. The cytological brush used for brush cytology sampling is in disposable, sterile packaging (Cervical Rambrush Type 1, Jiangsu Yada Technology Group, Yangzhou Jiangsu, China) and has a length of 190 mm.^21^ The order of sampling the different areas in the oral cavity is shown in Figure 1.

#### HPV and EBV infection detection with polymerase chain reaction (PCR)

Sample processing and detection of HPV- and EBV-specific sequences were performed at the Institute of Metagenomics, University of Debrecen, Hungary.

For the detection of HPV infection, exfoliated cells collected with the cytology brush were placed in ThinPrep Pap Test with PreservCyt solution (Hologic, Marlborough, Massachusetts, USA) and samples were stored at –20°C until processing. DNA extraction from exfoliated cells was performed as follows: the 20 ml sampling solution with mucosal cells was centrifuged at 2500 rpm for 10 min at room temperature, the supernatant was drained, and the pellet was resuspended in 200 μl of phosphate-buffered saline (PBS) solution. The DNA extraction from the 200 μl cell suspension was performed by the Viral DNA/RNA Extraction Kit (BioTeke Corporation, Wuxi, China) using the BioTeke automated magnetic isolation system following the manufacturer’s recommendation.

The quality of extracted DNA was checked using β-globin PCR. HPV-specific sequences were detected with MY09/MY11-GP5+/GP6+ consensus nested PCR specific to the highly conserved, approximately 450 basepair-long fragments of the L1 open reading frame (ORF) in the mucosal HPVs.^28^

For HPV genotype identification by the sequencing of MY09/11 and/or GP5+/6+ amplimers; PCRs were performed with Phusion High-Fidelity DNA Polymerase (Thermo Scientific, Waltham, USA); amplimers were purified by QIAquick Gel Extraction Kit (Qiagen, Hilden, Germany) and were sequenced in duplicates from both directions using the dideoxy chain termination method (Macrogen, Amsterdam, The Netherlands). Sequences were analysed against the HPV reference genomes available in the papillomavirus episteme (PaVe) database^23^ using CLC Main Workbench 7.9.1 (Qiagen, Aarhus, Denmark). HPV-16 positivity was examined by a type-specific primer set using oligonucleotide primers specific for the E7 ORF of the most frequent HPV genotype.^8^

Similarly to HPV detection, nested PCR amplifying a 97-basepair-long region of the internal repeat of the BamH1-W fragment of the viral genome was used to demonstrate the EBV genome in the clinical specimens. Briefly, primers EBV-F (5’-GAGACCGAAGTGAAGGCCCT-3’) and EBV-R (5’-ACAGCTCCTAAGAAGGCACC-3’) were used to amplify a 171-basepair-long amplicon, and then the amplification with primers EBV B-F (5’-GCCAGAGGTAAGTGGACTTT-3’) and EBV B-R (5’-GAGGGGACCCTGAGACGGGT-3’) resulted in a 97-basepair-long fragment within the 25 µL containing 1× DreamTaq Green Master Mix (Thermo Scientific, Waltham, USA), 25 pmol of each primer and 2 µL (0.1–0.3 µg) template DNA. Thermal profile was the following: 94°C for 5 min; 35 cycles of 94°C for 30 s, 58°C for 30 s, and 72°C for 30 s; and a final extension at 72°C for 5 min in both rounds. DNA from the EBV-positive B95-8 cell line was used as a positive control.^16^

#### Statistical analysis

For all groups, data were expressed as the mean ± the standard deviation (SD). The distribution of variables was evaluated by Kolmogorov–Smirnov test. A two-sample t-test was used for the age and Mann–Whitney test for unstimulated whole salivary flow rate comparison, while Pearson’s Chi-square test and Fisher’s exact test were used to determine any difference in the HPV and EBV infection rate between the healthy control (1), xerostomia (2), hyposalivation (3), and Sjögren’s syndrome (4) groups. SPSS software (version 15.0 SSP) was used for analysing all data. Results were considered significant at p < 0.05. Power determination was performed with the G-Power 3.1.9.2 Software (Heinrich Heine University, Düsseldorf, Germany).

## RESULTS

Out of the 60 patients, 10 (16.7%) had confirmed SS based on the ACR-EULAR classification criteria. According to the questionnaire, 53 (88.3%) patients felt subjective oral dryness (xerostomia), and 26 (43.3%) patients had measurable hyposalivation (UWS flow rate ≤ 0.1 ml/min).

Among the healthy control group patients there were no abnormal sicca symptoms mentioned in the questionnaire. During the clinical examination, no oral mucosal lesions were found in the healthy control group. Among the 60 patients, oral lichen planus was found on the buccal mucosa (confirmed by histopathology of one patient), and in another patient, oral aphthous lesions were scattered throughout the oral mucosa. The mean age of the 60 patients was 56.7 ± 15.6 years, and the male–female ratio was 1:9. In the healthy control group, the mean age was 49.7 ± 8.7 years and the male-female ratio was 1:9.

According to the diagnostic outcomes, patients and healthy controls performed in four groups: (1) healthy controls (n = 32), (2) xerostomia group (without hyposalivation and Sjögren’s syndrome, n = 28), (3) hyposalivation without Sjögren’s syndrome (n = 22), and group (4) patients with diagnostically proven primary Sjögren’s syndrome (n = 10).

### Sialometry Results

**Fig 2:**
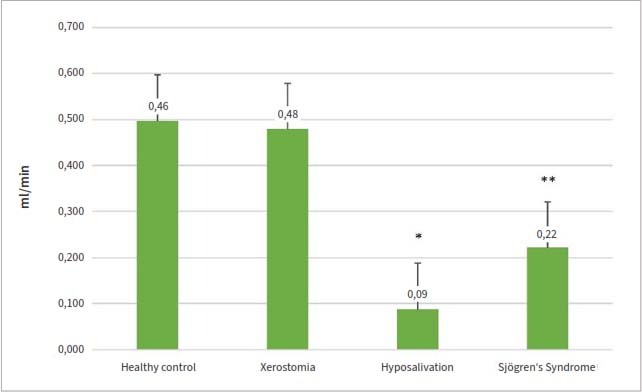
Unstimulated whole saliva flow rate of patients with dry mouth and/or primary Sjögren’s syndrome (n = 60) compared to healthy controls (n = 32).

Among the patients UWS flow rate was 0.29 ± 0.31 ml/min, while in the healthy control group it was 0.46 ± 0.27 ml/min. Obtained data revealed a statistically significant difference between controls (group 1) and the hyposalivation group (group 3) (0.09 ± 0.04 ml/min; p < 0.001, respectively) and between controls (group 1) and SS patients (group 4) (0.22 ± 0.21 ml/min; p = 0.006, respectively) (Fig 2).

### Histopathological Results of the Minor Salivary Gland Biopsy

**Fig 3:**
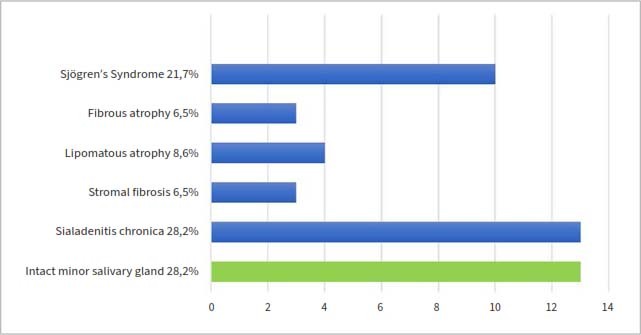
Histopathological diagnoses of the minor salivary gland biopsy samples.

Of the 60 patients, 46 underwent lower lip biopsy to verify primary Sjögren’s syndrome. Histopathological diagnosis was intact minor salivary gland in 28.2% (13), chronic sialadenitis in 28.2 % (13), stromal fibrosis in 6.5 % (3), lipomatosus atrophy in 8.6% (4), fibrosus atrophy in 6.5% (3 patients) and SS in 21.7% (10 patients) of the examined subjects (Fig 3). HPV infection did not cause statistically significantly more histological abnormalities in the minor salivary glands according to the biopsy evaluation results. Ratios concerning minor salivary gland alterations with oral mucosal HPV infections are shown in Table 1.

**Table 1:** HPV and EBV infection positivity percentage in the healthy controls and different patient groups

Subjects (n = 92)	Ratio of histopathological alterations in the non-HPV and non-EBV-positive cases	Number and ratio of HPV-positive cases	HPV genotypes (numbers of cases)	Ratio of histopathological alterations in the salivary gland samples of the HPV-positive cases (n = 5)	EBV-positive cases (n = 43)	Ratio of histopathological alterations in the salivary gland samples of the EBV-positive cases (n = 43)
Controls (n = 32)	–	1 (3.12%)	HPV-16+ (1)	–	14 (43.7%)	–
Xerostomia (n = 28)	Atrophy: 7 (28%) chronic sialadenitis: 2 (8%)	3 (10.7%)	HPV-16+ (1) HPV-11 MY+, GP+ (1) HPV-33 GP+ (1)	Atrophy: 1 (33%) chronic sialadenitis:1 (33%)	17 (60.7%)	Atrophy: 3 (17.6%)
Hyposalivation (n = 22)	Atrophy: 4 (21.0%) chronic sialadenitis: 5 (26.3%)	2 (8.26%)	MY+, GP+ (2)	0	6 (28.5%)	Fibrosis: 2 (33.3%) Atrophy: 1 (16.6%) Chronic sialadenitis: 1 (16.6%)
Sjögren’s syndrome (n = 10)	Positive focus score: 11 (100%)	0	–	–	6 (54.5%)	Positive focus score: 6 (100%)

DNA – deoxyribonucleic acid; EBV – Epstein–Bar virus; HPV – human papillomavirus; MS – multiple sclerosis; ORF – open reading frame; PaVe – papillomavirus episteme; PBS – phosphate-buffered saline; PCR – polymerase chain reaction; RNA – ribonucleic acid; SD – standard deviation; SLE – systemic lupus erythematosus; SS – Sjögren’s syndrome; UWS – unstimulated whole saliva.

### The Prevalence of HPV in the Different Patient Groups

Based on the β-globin PCR, all samples contained sufficient amounts of amplifiable DNA, and no PCR inhibitor was present in the isolated nucleic acid. All samples were suitable for further analysis.

Of the 60 patients with dry mouth, HPV-specific sequences were detected in samples from five patients (8.3%). One patient had HPV-16 positivity by type-specific PCR with GP positivity in the xerostomia patient group, while Sanger sequencing confirmed HPV-11 and HPV-33 positivity in two additional samples; HPV-11 positivity occurred in the hyposalivation group with MY/GP positivity; and HPV-33 positivity occurred in the xerostomia patient group with GP positivity. HPV-33, like HPV-16, is high-risk, while HPV-11 is a low-risk genotype. The remaining two samples (3.3%) were weakly HPV positive, but due to the low viral copy number in the samples genotyping was not successful either by virus-specific PCR or Sanger sequencing. No HPV infection occurred in the SS patient group. In the healthy control group, one patient (3.1%) showed HPV 16 positivity. Neither the positivity between the patient groups was statistically significantly different, nor was there a statistically significant difference in oral mucosal HPV-infection prevalence between the patient groups and the healthy controls by the Fisher’s exact test (Table 1). In a few cases, HPV genotyping was unsuccessful due to the low number of copies. These cases were classified as slightly positive, and not identifiable. The β-globin-specific PCR yielded in all samples tested.

### Prevalence of Epstein–Barr Virus Infection in the Different Patient Groups and Controls

**Fig 4:**
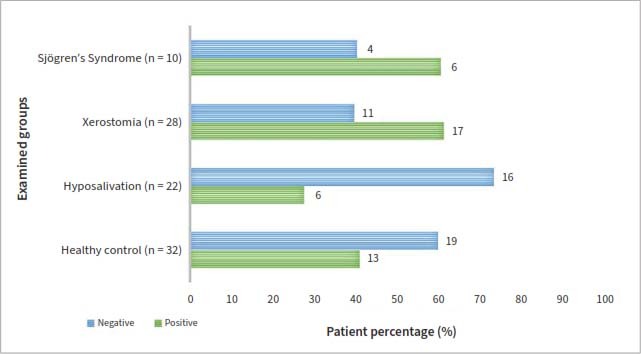
Prevalence of Epstein–Barr viral infections in the different patient groups with dry mouth and/or Sjögren’s syndrome compared with healthy controls.

EBV was detected in oral mucosa samples of 29 patients (29/60, 48.3%) with dry mouth and/or SS, 17 in the xerostomia group, 6 in the hyposalivation group, 6 in patients suffering from SS. In the healthy control group, EBV-specific sequences were detected in samples from 14 individuals (43.8%). Ratios of EBV-positive cases in the different patient groups and their relations to the different minor salivary gland biopsy findings are shown in Table 1 and Figure 4. Neither the positive ratio nor oral mucosal EBV infection prevalence were significanty different between the patient groups and the healthy controls by the Pearson's Chi-square test.

## DISCUSSION

Sixty patients with orofacial sicca symptoms and/or primary Sjögren’s syndrome were examined and compared with the data of 32 age- and sex-matched healthy controls in this present cross-sectional study. It is important to emphasise that this study was established to find orofacial mucosal viral HPV or EBV infections as causative factors in dry mouth or Sjögren’s syndrome.

### HPV Infections in Dry Mouth, Sjögren’s Syndrome and Healthy Controls

The results revealed that out of the 92 patients, the orofacial mucosa was HPV-infected in 5 (5.4%). Its ratio did not differ from the outcomes of a previous Hungarian study where orofacial and, at the same time, genital mucosal HPV-infection were investigated.^30^ In that study, the positivity rate was 5%.

In this study’s four patient groups, the oral mucosal HPV infection rate was 3% in the healthy controls, 10% in the xerostomia group, and 8% in the hyposalivation group, but 0 in Sjögren’s syndrome. Thus, in patients with subjective or objective orofacial sicca symptoms HPV-infection rate of the oral mucosa was increased compared to the healthy controls and to the ratio in Sjögren patients. Nevertheless, neither xerostomia nor hyposalivation nor Sjögren’s syndrome were linked with an increased HPV infection compared to the healthy subjects’ rate in patients from the central region and the capital of Hungary. Until now no studies investigating dry mouth and HPV carriage could be found in the literature.

One study examined the association between HPV carriage and Sjögren’s syndrome. Chen et al. (2022), in their 12-year follow-up, investigated subjects with HPV viral infections from healthcare data provider sources compared to HPV-free controls. The ratio of HPV carriers in the Taiwanese population was 6.3%. Their subjects were free from Sjögren’s syndrome at the initial examination year. At the end, their results revealed a two-fold risk for SS in the HPV-infected populations to the end of the 12th year.^4^

In this present Hungarian investigation, all subjects were newly diagnosed: either with sicca syndrome (xerostomia and/or hyposalivation, xerophthalmia and/or keratoconjunctivitis sicca), and/or Sjögren’s syndrome, HPV and/or EBV viral infections. Therefore, the results are only partially comparable. It should also be noted that Chen et al do not explain their patient’s virus carriage identification source: whether it had been sampled from the head and neck or from the genital region.

### EBV Infections in Dry Mouth, Sjögren’s Syndrome and Healthy Controls

A more accurate picture of EBV infection and the status of infection in patients with dry mouth can be obtained by detecting viral genetic material and antigens. An increase in the amount of viral DNA or the appearance of EBV-specific proteins clearly indicates the reactivation of the virus.

In a study by Saito and colleagues (1989), EBV DNA was detected in 18% of peripheral blood mononuclear cell samples from patients with Sjögren’s syndrome and 13% of salivary gland biopsies from healthy controls, whereas virus-specific sequences were present statistically significantly higher (78%) in tissue samples from patients.^26^

Several other studies have reported statistically significantlytly higher EBV DNA positivity in salivary gland biopsy specimens from patients with Sjögren’s syndrome compared to controls; depending on the sensitivity of the detection method used, the EBV genome was detected in 50 to 86% of salivary gland biopsies. However, the low EBV DNA positivity detected in the serum of patients with Sjögren’s syndrome suggests a tight immune systemic control of viral infection in the blood.^17^ Pflugfelder and colleagues (1990) detected a similarly high EBV positivity rate in lacrimal gland samples from patients with Sjögren’s syndrome compared to controls (80% vs 32%).^25^ As it is evident from the literature, most of the authors examined blood samples and salivary gland biopsy samples to detect EBV infection. According to our knowledge, there is no previous analysis regarding oral mucosal cell HPV or EBV infections as possible factors in xerostomia or in the development of salivary gland damage and primary Sjögren’s syndrome.

Our recent findings revealed that in oral mucosal cells, EBV viral DNA could be detected between 28% and 60% of the samples from patients with dry mouth and/or Sjögren’s syndrome, and 43% in the healthy controls. In summary, EBV positivity rate in the mucosal cells was not significantly different from the control group in any of the four patient groups tested, which is different from the results of previous publications that proved EBV’s role in primary Sjögren’s syndrome. It should be noted, that in this case, the presence of viral DNA has been in mucosal cells and not in salivary gland samples examined. The reason for the different outcomes might be that nucleic acid extracted from oral mucosal exfoliated cells may under-represent the actual EBV positivity of the salivary glands or the serum. It might be considered as a limitation of this analysis. A further study of both EBV and HPV might clarify how oral mucosal presence corresponds with blood and salivary gland test results. It is also possible that the virus disappears from the oral mucosa, but blood test and /or salivary gland examination might show the systemic viral DNA presence, as a possible factor in initiating a systemic autoimmune reaction described by Barcelos et al^2^ and others.^17,34^

### Histopathological Alterations in the Minor Salivary Glands Related to Mucosal HPV and/or EBV Viral Infections

Considering the histopathological findings of the minor salivary gland biopsies, most frequent alterations were atrophy (fibrous and lipomatous) besides the focal infiltration in Sjögren’s syndrome. Obtained data revealed that neither oral mucosal HPV nor EBV positivity statistically significantly influenced the number and type of alterations in dry mouth patients and/or in Sjögren’s syndrome (Table 1).

In a previous Hungarian study, Epstein–Barr virus prevalence was examined in oral squamous cell cancer and in potentially malignant oral disorders in an eastern Hungarian population in 2009 and 19.1% EBV positivity was found in the exfoliated cells.^16^ In our Central Hungarian patient group, EBV-specific sequences could be detected at 1.5–2 times that of the eastern Hungarian rate, while samples were evaluated by the same laboratory and experts. The authors propose that the reason might be a regional difference in the lifestyle of people: the central part is more industrialised, and the population density is higher compared to the rather agricultural and less populous eastern area. Therefore, people are less likely to get infected. This aspect, however, should be further examined.

It is also questionable if dry mouth might be a starting point for increased mucosal viral infections possibly leading to autoimmune disease. According to the obtained data oral mucosal viral infection rate was not higher in any of the dry mouth groups compared to the controls. Thus, the oral mucosal viral infection rate was not higher in any stages of dry mouth and/or Sjögren’s syndrome. This study could not prove oral mucosal HPV or EBV’s direct role in primary Sjögren’s syndrome in the examined Hungarian population. On the contrary, these viral infections cannot either be considered as results of increased mucosal susceptibility because of dry mouth and/or Sjögren’s syndrome. It should be noted that patients involved in this study were the whole number of examined persons with xerostomia in the central region of Hungary (2.9 million people) during the studied one-year period. A longer follow-up with more patients might reveal a stronger correlation with mucosal EBV or HPV viral infections and dry mouth and/or Sjögren’s syndrome in this central Hungarian region.

### Limitations

Limitations of this study were the low number of cases with Sjögren’s syndrome. It should be noted, however, that although Sjögren’s syndrome is one of the most common autoimmune diseases, it is still a rare disease (in Hungary). So, the number of new diagnoses was determined and limited by the sum of patients shown up at the sessions of the Xerostomia Clinic Working Group during the investigation period. Unfortunately, restrictions due to the COVID-19 pandemic made personal check-ups and clinical trials difficult at that time.

## CONCLUSIONS

Since ratios of HPV and/or EBV DNA rates in orofacial mucosal cells did not differ statistically significantly in patients with xerostomia, hyposalivation, or Sjögren’s syndrome compared to healthy controls, it cannot prove the provocative role of either the HPV or the EBV virus in our Hungarian patients with dry mouth and/or primary Sjögren’s syndrome.

Xerostomia and hyposalivation, as well as Sjögren’s syndrome, have not been accompanied by statistically significantly more alterations in the salivary gland biopsies of the HPV- and/or EBV-positive subjects, as these signs are most probably not in connection with the HPV or EBV infections of the oral mucosal cells. These alterations are similarly frequent in the virus-negative cases.
